# Insights into the Global and Mexican Context of Placental-Derived Pregnancy Complications

**DOI:** 10.3390/biomedicines13030595

**Published:** 2025-03-01

**Authors:** Erika Chavira-Suárez

**Affiliations:** 1 Unidad de Vinculación Científica de la Facultad de Medicina, Universidad Nacional Autónoma de México (UNAM) en el Instituto Nacional de Medicina Genómica (INMEGEN), Mexico City 14610, Mexico; erika@bq.unam.mx; 2 Departamento de Bioquímica de la Facultad de Medicina, Universidad Nacional Autónoma de México (UNAM), Mexico City 04360, Mexico; 3 Centro de Investigación en Ciencias de la Salud, Facultad de Ciencias de la Salud, Universidad Anáhuac México Campus Norte, Huixquilucan 52786, Mexico

**Keywords:** pregnancy complications, incidence, prevalence, trends, healthcare strategies, public policies

## Abstract

Placental-derived pregnancy complications encompass a range of disorders that hinder optimal fetal development, significantly impacting maternal and neonatal health outcomes. Key conditions include placental insufficiency, preeclampsia, fetal growth restriction (FGR) or intrauterine growth restriction (IUGR), fetal overgrowth, and gestational diabetes mellitus (GDM), which together contribute to a heightened risk of preterm birth, perinatal mortality, and long-term developmental challenges in affected infants. These complications are particularly notable because they generate approximately 80% of pregnancy disorders and pose significant public health concerns across diverse global contexts. Their management continues to face challenges, including a lack of consensus on diagnostic criteria and varied implementation of care standards. While imaging techniques like magnetic resonance imaging (MRI) and Doppler ultrasound have emerged as critical tools in clinical assessment, disparities in access to such technologies exacerbate existing inequalities in maternal and fetal health outcomes. Maternal and pregnancy care is a broad range of services aimed at promoting the well-being of women throughout the perinatal period. However, access to these services is often limited by economic, geographical, and sociocultural barriers, particularly for marginalized groups and women in low- and middle-income countries (LMICs). The implementation of targeted interventions designed to address specific obstacles faced by disadvantaged populations is a crucial component of bridging the gap in health equity in maternal care. Public health authorities and policymakers strive to develop evidence-based strategies that address the interplay between healthcare access, socioeconomic factors, and effective interventions in order to mitigate the adverse effects of placental-derived pregnancy complications. Continued research and data collection are essential to inform future policies and practices to improve outcomes for mothers and infants.

## 1. Introduction

The placenta is a vital temporary organ that develops during the first three months of pregnancy and expands in tandem with the growth of the uterus, serving as a life-sustaining mechanism for fetal development [1]. It is responsible for numerous crucial functions, among which are (1) the provision of essential nutrients to the fetus, facilitating the transfer of nutrients from the mother’s bloodstream to the fetal circulation [2], (2) the oxygen transmission from the maternal blood to the fetal blood, while simultaneously removing waste products, such as carbon dioxide, urea, and creatinine from the fetus [3], (3) the secretion of various hormones, including human chorionic gonadotropin (hCG), human placental lactogen (hPL), estrogens, and progesterone, which are crucial for regulating maternal metabolism and preparing the body for childbirth [4], and (4) the provision of fetal protection from certain infectious pathogen transmissions, as well as the transfer of maternal antibodies during the early stages of life [5].

**Figure 1:**
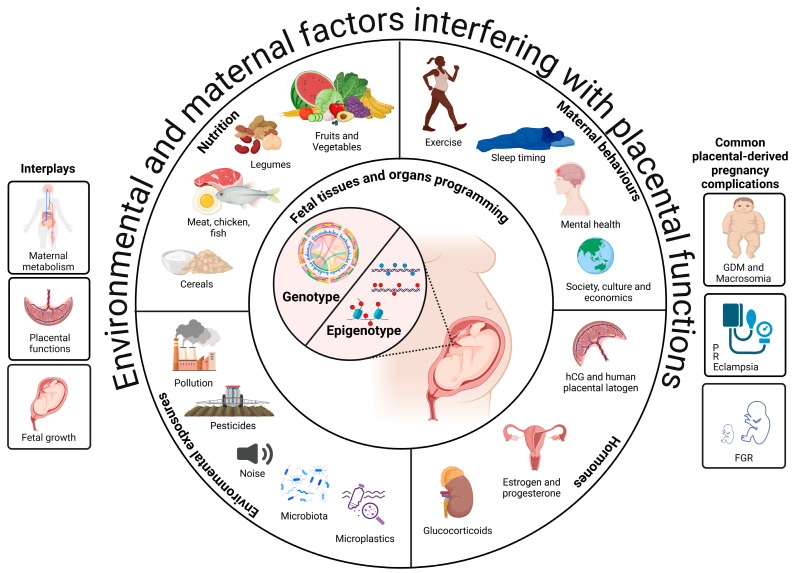
The intricate relationship between maternal metabolism, placental function, and fetal growth health. This scheme depicts the interplay between maternal metabolism, placental functions, and fetal growth as critical aspects for shaping the development of the fetus and influencing long-term health outcomes for both mother and neonate. Environmental factors, maternal nutrition and behaviors, and circulating maternal hormones interfere with components of the placental function that, consequently, impact fetal and neonatal health. During pregnancy, exposure to these different components can alter the genotype and epigenetic composition of the placenta, affecting its hormone production and creating a feedback loop that affects both nutrient availability and fetal health outcomes. The primary severe complications of pregnancy resulting from placenta dysfunction are DGM, preeclampsia, and FGR. These diseases have significant public health concerns across the globe and in diverse contexts. Abbreviations: hCG, human chorionic gonadotrophin; FGR, fetal growth restriction; GDM, gestational diabetes mellitus. Created in https://BioRender.com (access date: 3 January 2025).

Several maternal factors, including hormonal and nutritional statuses, environmental exposures, and genotypes and epigenotypes, interfere with placental functions, which can have a significant impact on fetal outcomes [6,7] (see Figure 1). Pregnancy complications arising from abnormal placental function can lead to significant maternal and fetal health issues in the short and long terms [8]. In this timing sense, abnormalities in placental function can lead to various pregnancy complications in relation to placental insufficiency, maternal metabolism, and fetal growth, which have significant long-term health implications for both the mother and the newborn [6,9].

Long-term studies on placental disorders have increasingly demonstrated that the outcomes of pregnancy complications have an impact on the health of both mothers and their newborns [10]. For instance, women who experience preeclampsia or GDM are at a heightened risk for chronic health conditions, including cardiovascular disease, hypertension, cerebrovascular events, and metabolic disorders, later in life [11]. In a similar manner, neonates who have been exposed to preeclampsia exhibit a significant predisposition to cardiovascular diseases and metabolic issues as young adults [12]. These phenomena underscore the concept of developmental programming, which suggests that certain prenatal conditions can lead to chronic health issues later in life, regardless of genetic predisposition. In conjunction with this concept, the Barker hypothesis proposes that prenatal stressors, such as maternal malnutrition or psychological stress, may result in an elevated risk of metabolic syndrome and related diseases in offspring, thereby establishing a cycle of health challenges that may impact maternal health as well [13]. Additionally, exposure to obesity and a high-fat diet during pregnancy has been linked to adverse outcomes, such as increased appetite in offspring and a predisposition to obesity and metabolic diseases [10]. The importance of maternal nutrition cannot be overstated; inadequate intake of micronutrients can adversely impact fetal growth and development, particularly in low-income groups where access to nutrient-dense foods may be restricted [14,15].

Research on placental dysfunction has predominantly focused on high-income countries (HICs), resulting in a disparity in comprehension among LMICs. Placental disorders are linked to higher rates of maternal and perinatal mortality in LMICs, with over 95% of these deaths occurring in these regions. Factors such as malnutrition, inaccessibility to healthcare, and infectious diseases compound the hazards associated with placental dysfunction [16].

Health equity refers to the principle that all individuals should have fair access to quality healthcare, resulting in equal health outcomes across different population groups [17]. The achievement of health equity remains a significant global challenge, particularly in the field of maternal and pregnancy-related care, where disparities are evident. Evidence suggests that healthcare inequities disproportionately impact women, especially those who are pregnant or birthing, as well as their infants and families [18]. Despite the advancements in healthcare policies, substantial disparities persist, particularly among underrepresented groups, commonly referred to as underreported (U3) populations. These disparities lead to elevated rates of maternal mortality and morbidity, as well as adverse health outcomes for infants, largely attributed to social determinants of health, such as poverty, discrimination, and inadequate healthcare resources [19]. Placental disorders further complicate the landscape of maternal health, encompassing diverse conditions that have the potential to adversely affect pregnancy outcomes [6,7].

In order to reduce pregnancy complications related to placental dysfunction, with the most common and high prevalence in LMICs, it is crucial to consider local and regional health determinants. The aim of this review is to focus on the impacts of placental dysfunction-related pregnancy complications in a global and Mexican context, discussing public policies, emerging insights, technological advances, gaps in research, and future directions.

## 2. Pregnancy Complications with a High Prevalence in Human Populations Related to Placental Dysfunction

Placental abnormalities are conditions in which the placenta does not develop or function properly during pregnancy. These abnormalities can arise at any stage of placental development and may significantly impact both maternal and fetal health. They can disrupt essential functions of the placenta, leading to various complications during pregnancy and delivery. Placental anomalies, such as placental previa, placental accreta spectrum, placental abruption, vasa previa, chorioangioma, and choriocarcinoma, are distinguished by distinctive characteristics, with a prevalence ranging from 0.3% to 2% among populations [20]. Although the identification, monitoring, and treatment of placental abnormalities during pregnancy pose significant challenges, the present review will solely focus on pregnancy complications with a high prevalence.

Pregnancy complications, which are prevalent among pregnant women, are frequently associated with placental anomalies, resulting in distinct risks and management challenges. Complications such as FGR, fetal overgrowth, small and large for gestational age (SGA and LGA, respectively), preeclampsia, GDM, and preterm birth, among others, are the main pregnancy complications that have significant long-term health implications for both the mother and the newborn [6,9]. Understanding the interplay between maternal metabolic status, placental function, and fetal growth is crucial for improving pregnancy and neonatal outcomes.

### 2.1. Maternal Metabolic Status-Associated Disorders

Maternal metabolism throughout pregnancy undergoes significant adaptations, including increased insulin resistance, altered fat metabolism, and modifications in glucose homeostasis (normal physiological responses), ensuring an adequate supply of glucose to the fetus. In this regard, a healthy metabolic state is essential for optimal placental function and fetal development [21].

For instance, maternal obesity can lead to an increased risk of GDM, characterized by glucose intolerance diagnosed between 24 and 28 weeks of gestation [22]. It poses risks not only to the mother, including an increased likelihood of developing type 2 diabetes postpartum [23], but also to the fetus (overgrowth), leading to structural and functional changes in the placenta, including delayed maturation, inadequate nutrient and oxygen transfer to the fetus, and involving a combination of oxidative stress and inflammatory responses. These alterations may result in severe complications such as macrosomia and metabolic disorders later in life [8]. The implications of these disorders are not only in the placenta but extend beyond the immediate postpartum period, and they can lead to a cycle of obesity and metabolic disease across generations, with fetal exposure to an unhealthy intrauterine environment playing a critical role in developmental programming [23].

### 2.2. Placental Insufficiency-Associated Disorders

Abnormal placental function can lead to conditions such as FGR (also known as IUGR), preterm birth, and preeclampsia, which are severe complications associated with adverse pregnancy outcomes [24]. FGR, which is used to define fetuses with an estimated fetal weight that is less than the 10th percentile for gestational age, can arise from various factors affecting the placenta, umbilical cord, and maternal (hypertension and diabetes) or fetal (genetic disorders or congenital anomalies) conditions [9,25]. Maternal hypertension and inadequate placental blood flow are factors also related to preterm birth [26]. Preeclampsia is another complication associated with placental insufficiency that shares some pathophysiological traits with FGR, as both conditions can stem from inadequate remodeling of maternal blood vessels supplying the placenta [24].

Research indicates that maternal health prior to conception, including nutritional status and metabolic health, influences placental development and function [1]. Some of the causes of placental dysfunction or placenta insufficiency are in connection with vascular remodeling issues, maternal chronic diabetes, hypertension, and autoimmune disorders, as well as lifestyles factors such as smoking and drug use. In connection, the few observable symptoms reported by healthcare providers include decreased fetal movement and smaller-than-average fundal height measurements [27].

### 2.3. Fetal Growth-Associated Disorders

Fetal growth is influenced by the interplay between maternal metabolic status and placental function (Figure 1). Adequate nutrient supply from the mother through a well-functioning placenta is necessary for proper fetal development [25]. On the contrary, fetal growth disorders may arise from a variety of etiological factors, such as placental insufficiency, maternal health conditions such as hypertension and diabetes, inadequate nutrition, substance abuse, infections, and genetic abnormalities [9,25]. The timing of delivery is critical, particularly in cases of FGR, as it must balance the risks of prematurity and stillbirth [26].

Fetal growth disorders, including SGA and LGA, arise when a fetus fails to meet expected growth standards during pregnancy, resulting in complications such as increased risks of delivery challenges and neurodevelopmental and metabolic issues. The term SGA refers to newborns whose birth weight is less than the 10th percentile for their gestational age, compared to the term LGA that refers to those whose birth weight exceeds the 90th percentile [28]. Both SGA and LGA infants may face challenges during delivery, such as necessitating cesarean sections or experiencing birth injuries. Additionally, they may encounter complications such as feeding difficulties, temperature instability, and elevated risks of subsequent health issues [28,29].

Studies have shown that interventions aimed at improving maternal nutrition can lead to better pregnancy outcomes, particularly in populations with high rates of undernutrition. Furthermore, maternal education on nutrition is vital for ensuring that women understand the importance of dietary choices during pregnancy [30].

## 3. Global Versus Mexican Impact of Placenta-Derived Pregnancy Complications

The relevance of pregnancy complications is underscored by rising maternal mortality rates in various regions, particularly in the United States, where the maternal mortality rate has more than doubled from 1987 to 2018, reaching 17.4 deaths per 100,000 live births. Globally, the maternal mortality ratio of pregnancy complications has declined by 38% between 2000 and 2017 [30]. However, most maternal deaths still occur in LMICs, where access to quality healthcare remains limited [31]. Racial and geographic disparities are significant; black women are three times more likely to die from pregnancy-related causes compared to white women, highlighting systemic inequities in healthcare access and outcomes [32]. Understanding the mechanisms behind placental function is crucial for improving pregnancy outcomes and reducing the risks associated with placental-related disorders.

### 3.1. Impact of Placental-Derived Pregnancy Complications in the Globe

The incidence of pregnancy complications is influenced by various factors, including socioeconomic status, access to healthcare services, and environmental conditions [31]. In the U.S., over 60,000 birthing individuals experience severe maternal morbidity annually, with a notable rise in such cases in recent years [30]. The current situation regarding pregnancy complications is alarming in that the implications of placental insufficiency extend beyond individual pregnancies, affecting public health systems globally [8].

Around the globe, ten million women develop preeclampsia annually, resulting in significant morbidity and mortality. The global burden includes an estimated 76,000 maternal deaths and 500,000 infant fatalities each year attributed to hypertensive disorders of pregnancy, including preeclampsia. In Latin America, preeclampsia is recognized as the leading cause of maternal mortality [33].

The management of complications arising from abnormal placental function contributes significantly to healthcare expenditure. Increased rates of neonatal intensive care unit (NICU) admissions for FGR and LGA infants elevate costs [6]. Children who experience FGR are at a higher risk for stillbirth, neonatal complications, cardiovascular diseases, and metabolic disorders. LGA infants face risks during delivery, including birth trauma and increased likelihood of cesarean delivery. They are also at risk for metabolic syndrome costs later in life [25]. The prevalence of these conditions can lead to broader societal challenges, including workforce productivity losses and increased healthcare demands as affected individuals age [34].

Abnormalities in placental function can lead to conditions such as FGR or fetal overgrowth, each associated with increased morbidity and mortality. FGR is characterized by the failure of the fetus to reach its genetically determined growth potential, primarily due to placental insufficiency [25]. This condition affects 5–10% of pregnancies and is linked to various maternal factors, including poor nutrition, smoking, and pre-existing health conditions like hypertension. In developed countries, the prevalence tends to be lower due to better prenatal care compared to developing regions, where it can be significantly higher [35]. Conversely, fetal overgrowth can occur due to maternal obesity, diabetes, or excessive gestational weight gain [36]. The incidence of LGA has been rising in recent years, correlating with increasing rates of maternal obesity [25]. Current estimates suggest that LGA affects about 10–15% of pregnancies, with ongoing research indicating a potential rise in prevalence linked to lifestyle changes and dietary habits [36].

A systematic review of 25 studies involving 2895 pregnancies complicated by severe early-onset FGR found that the antenatal mortality rate was approximately 12%. Specifically, 355 fetuses died antenatally out of 2895 pregnancies. The neonatal mortality rate was reported at about 8%, with 192 live-born neonates dying in the neonatal period. Overall, 81% of pregnancies complicated by early-onset FGR survived to birth. However, long-term follow-up indicated that about 12% of survivors experienced neurodevelopmental impairment or cerebral palsy [37].

Pregnancies complicated by fetal overgrowth (often linked to conditions like gestational diabetes) have been associated with increased perinatal morbidity and mortality. Specific mortality rates for fetal overgrowth were not detailed in the sources, but it is known that such conditions elevate risks for complications during delivery and long-term health issues for the offspring. Children born from pregnancies with fetal overgrowth are at higher risk for developing obesity, diabetes, and cardiovascular diseases later in life [25].

### 3.2. Impact of Placental-Derived Pregnancy Complications in Mexico

The incidence of fetal overgrowth, specifically referred to as fetal macrosomia (FM), in Mexico has been assessed in several studies. Historically, the prevalence of FM in Mexico has varied. A previous estimate from 2004 to 2005 indicated a prevalence of 3.8%, which aligns more closely with recent findings than earlier studies that reported higher rates, such as an 18.6% incidence observed in a hospital study in Saltillo. In 2020, the national incidence rate of macrosomia is approximately 2.75%, with a notable difference between genders; it is higher in males at 3.17% compared to 2.31% in females. The rates can vary by state, with some regions reporting higher incidences of LGA births. The states with the highest incidences were Sonora (6.2%), Baja California Sur (5.44%), and Sinaloa (5.36%). In contrast, Mexico City reported the lowest incidence at 1.28% [38]. Several factors contribute to fetal overgrowth, including maternal obesity and gestational diabetes, which are increasingly prevalent in Mexico [22]. Socioeconomic factors also play a role, as certain populations, such as the Maya community, exhibit different growth patterns influenced by their sociodemographic conditions [39].

The incidence of preeclampsia in Mexico is a significant public health concern, with estimates suggesting a prevalence ranging from 10% to 14% according to the World Health Organization (WHO) [40]. Preeclampsia is responsible for approximately 4000 maternal deaths annually in Mexico, highlighting its severity as a health issue. Among reported cases, about 94% are classified as mild preeclampsia, while severe cases account for approximately 3.75%, and eclampsia for about 1.75% [41]. Other studies show a prevalence that can reach up to 16.7%, indicating significant variability depending on the population studied and the methodologies employed [42]. The prevalence rates are influenced by several factors, including access to healthcare, socioeconomic conditions, and the presence of risk factors such as obesity. For instance, obesity is identified as a significant risk factor for developing preeclampsia, with women having a body mass index (BMI) ≥ 30 kg/m^2^ being at higher risk [33].

The prevalence of GDM in Mexico is generally estimated to be between 10% and 12% based on various studies; this figure may not fully capture the national prevalence due to insufficient data [43]. A recent study reported a prevalence of 44.5% using the International Association of Diabetes and Pregnancy Study Groups (IADPSG) criteria and 37.5% using the National Institute for Health and Care Excellence (NICE) criteria in a cohort from Northeast Mexico [44]. A survey among healthcare practitioners indicated a clinician-reported prevalence of 23.7%, reflecting regional differences in GDM incidence across Mexico [45]. This finding indicates that the choice of diagnostic criteria can significantly affect reported prevalence rates.

In another instance, a national retrospective study indicated that approximately 28.8% of adolescents in certain regions of Chiapas experienced FGR, highlighting a substantial prevalence among vulnerable populations [46]. In Yucatan, a study found the prevalence of FGR to be 9.06%, with higher rates among the Maya population at 12.4% [26]. For instance, Baja California Sur reported an LGA prevalence of 16.8% [47].

The incidence of FGR varies significantly across different regions and populations in Mexico. Recent studies indicate that approximately 28.8% of infants experience FGR, which is a specific type of FGR characterized by low birth weight (LBW). A study in Chiapas found that 12% of the sample had LBW, with FGR being prevalent among adolescents from indigenous communities. This suggests a strong correlation between socioeconomic factors and fetal growth outcomes [46]. In Guanajuato, the leading causes of infant mortality are conditions originating during the perinatal stage, with a sizable portion attributed to congenital malformations and chromosomal abnormalities. The region has reported high antenatal care coverage, yet disparities remain for marginalized populations [48]. Regional research indicates that FGR is more common among Maya women compared to non-Maya women, highlighting the influence of ethnic and socioeconomic conditions on fetal growth [39].

The perinatal mortality rate has been historically underreported in Mexico, particularly concerning stillbirths [49]. The linkage of live birth and death records from 2008 to 2019 revealed that neonatal mortality rates were significantly higher for preterm births compared to term births, as well as for SGA infants compared to those appropriate for gestational age (AGA). However, LGA infants did not show an increased mortality risk compared to AGA infants [50]. In 2020, Mexico reported 22,637 fetal deaths, with a considerable proportion attributed to conditions related to FGR. The study emphasized that conditions originating during the perinatal stage are major contributors to infant mortality [48]. The State of Mexico has been identified as having particularly high rates of morbidity and mortality related to preeclampsia, emphasizing the need for targeted public health interventions in this region [41].

## 4. Impact of Public Policies on Placental Dysfunction Disorders

Public policies related to maternal and fetal health significantly influence neonatal growth and development. These policies encompass a range of healthcare practices, monitoring protocols, and regulations regarding environmental exposures [30]. The following sections outline key areas where public policies are affecting fetoplacental and neonatal growth.

### 4.1. Maternal Health Monitoring

Several guidelines, such as those from the Royal College of Obstetricians and Gynaecologists (RCOG), emphasize the importance of accurately assessing fetal growth through ultrasound measurements and Doppler assessments. This is crucial for identifying SGA fetuses and managing conditions like FGR effectively [51].

In the UK, the National Health Service (NHS) has implemented the ’Saving Babies’ Lives’ Care Bundle initiative aiming to reduce stillbirth rates by 50% by 2025. It highlights evidence-based practices to improve outcomes, particularly for high-risk pregnancies [52]. This policy reflects an increasing focus on targeted interventions that can enhance fetoplacental health.

### 4.2. Environmental Exposures and Endocrine Disruptors

Air pollution has emerged as a critical risk factor, with multiple studies indicating that exposure to particulate matter (PM2.5, PM10, etc.) can elevate blood glucose levels in pregnant women. Findings from several studies demonstrate a significant association between air pollution and abnormal glucose tolerance during pregnancy, highlighting the need for attention to air quality as a component of prenatal care [53]. For example, a large cohort study found that increased levels of ambient air pollutants correlated with adverse glycemic outcomes [54].

Ambient temperature is another significant environmental factor affecting glycemic control during pregnancy. Increased environmental temperatures have been associated with maternal beta-cell dysfunction and elevated blood glucose levels, suggesting that extreme weather conditions may exacerbate the risk of GDM. In fact, rising temperatures have been consistently linked to higher rates of GDM, with studies indicating that temperatures exceeding 25 °C could lead to detrimental glycemic effects [55].

Exposure to endocrine-disrupting chemicals (EDCs) such as bisphenol A (BPA), persistent organic pollutants (POPs), heavy metals, and phthalates during pregnancy has been linked to adverse outcomes in fetoplacental development, including altered placental function and fetal growth metrics [6]. Elevated levels of arsenic in maternal blood or meconium have been linked to an increased risk of GDM. Other metals such as nickel, antimony, cobalt, and vanadium also show positive associations with GDM risk [54]. Public policies that regulate or limit the use of these chemicals in consumer products can significantly impact maternal environments and subsequently fetoplacental health.

### 4.3. Healthcare Accessibility and Quality

Policies that ensure equitable access to prenatal care are vital. Disparities in healthcare access can lead to higher rates of stillbirth and complications among marginalized communities. For instance, stillbirth rates are disproportionately higher among Black and Asian women in the UK compared to their white counterparts [34]. In LMICs, there is a pressing need for innovative monitoring technologies to accurately assess fetoplacental health, both in clinical settings and remotely. Current tools have limitations that hinder timely interventions during high-risk pregnancies [31].

On other hand, maternal nutrition plays a critical role in placental development. Policies promoting adequate maternal nutrition, including iron supplementation during pregnancy, are essential, as deficiencies can lead to placental dysplasia and affect fetal growth [52].

The significance of maternal vaccination is also pivotal in safeguarding both mothers and infants from infectious diseases during pregnancy and early life. Vaccines such as the inactivated influenza vaccine (IIV) and the Tetanus, Diphtheria, and Pertussis (Tdap) vaccine have been recognized for their safety and efficacy in pregnant women [56]. Vaccination has the potential to mitigate the risks associated with viral pathogens, as unvaccinated pregnant women exhibit higher rates of severe illness, hospitalization, and complications in comparison to their vaccinated counterparts. This holds particular significance for outbreaks such as influenza and SARS-CoV-2, which have been associated with heightened maternal mortality and adverse fetal outcomes, including placental lesions and fetal mortality in severe cases [57].

### 4.4. Future Policy Directions

There is a need for continued research into the mechanisms of placental function and the relationship with fetal growth. This includes exploring novel therapeutic strategies aiming to restore normal growth patterns in fetuses affected by placental dysfunction. Effective public health policies should integrate findings from ongoing research to implement guidelines supporting maternal health and fetal monitoring practices. Such policies may involve developing standardized protocols for monitoring fetal growth and managing pregnancies at risk for FGR or overgrowth. Enhancing training for healthcare providers on the latest monitoring techniques and interventions would improve outcomes in pregnancies complicated by abnormal fetal growth.

Access to healthcare is recognized as a universal social right; however, the reality in LMICs often contrasts sharply with that in HICs due to limited health resources that necessitate careful rationing. This situation raises critical issues regarding the equity and efficiency of healthcare systems in these regions [58]. For instance, while HICs generally have more substantial healthcare budgets, leading to better health outcomes, LMICs struggle with inadequate funding and infrastructure, which hampers their ability to address placental disorders effectively. The contrasting experiences of HICs and LMICs underscore the necessity for tailored policy interventions that consider each country’s unique context and healthcare challenges. For effective health financing reforms, it is crucial to address the specific performance issues within each country’s health financing arrangements [59]. By learning from successful models in HICs and adapting them to the realities of LMICs, policymakers can aim to enhance access, efficiency, and ultimately, the outcomes for mothers and infants affected by placental disorders.

## 5. Emerging Insights into Placental Function

Emerging insights into placental function reveal significant advancements in understanding the mechanisms and genetic factors influencing placental development, which are crucial for fetal health. Recent studies highlight various aspects of placental function, genetic influences, and technological innovations in research.

### 5.1. Genetic Influences on Placental Growth

A groundbreaking study conducted by researchers from Copenhagen University Hospital and other institutions identified 40 genetic variations associated with placental growth. This genome-wide association study involved a large cohort of approximately 65,000 children and highlighted that variations in the fetal genome are pivotal for placental weight. Notably, faster placental growth was linked to an increased risk of preeclampsia and earlier delivery, suggesting that an imbalance between this two factors are crucial. These findings underscores the placenta’s essential role as a mediator of maternal–fetal interactions and its impact on pregnancy outcomes [36].

Nutrigenomics is a burgeoning scientific field that investigates the complex interactions between nutrition and gene expression, particularly focusing on how dietary components can influence genetic pathways and overall health outcomes. This discipline is especially significant in the context of fetal health and placental disorders, as maternal nutrition directly affects both fetal development and pregnancy outcomes [60]. The causal mechanisms underlying these associations remain partially unresolved, particularly concerning the relative contributions of maternal and fetal genetic effects. Research should focus on identifying the nutritional components that enhance placental health and prevent associated disorders, paving the way for targeted interventions. By addressing these key areas, future research in nutrigenomics will contribute significantly to enhancing fetal health and preventing placental disorders, ultimately improving maternal and child health on a global scale [61].

### 5.2. Hypoxia and Placental Function

Another significant insight pertains to the role of hypoxia-inducible factors (HIFs) in placental function. Research indicates that chronic activation of HIF-2α can impair placental development, contributing to conditions such as FGR and preeclampsia. Specifically, increased levels of HIF-2α were observed in pathological placentas, while silencing this factor enhanced trophoblast differentiation and placental growth factor availability. This suggests potential therapeutic avenues for addressing placental dysfunction [62].

### 5.3. Oxygenation and Fetal Brain Development

The importance of oxygenation levels in the placenta has also been emphasized in recent studies. Research shows that adequate oxygenation during the third trimester is crucial for cortical brain development in the fetus. Insufficient oxygen can hinder cognitive outcomes later in life, linking placental health directly to neurological development. This connection was established through advanced imaging techniques that provided a clearer understanding of how placental conditions affect fetal brain growth [63].

### 5.4. Role of Biomarkers in Diagnosis

The clinical application of biomarkers to diagnose placental dysfunction remains an evolving field. However, the role of biomarkers in the diagnosis and management of placental dysfunction, particularly in conditions such as GDM, preeclampsia, and FGR, has gained considerable attention in recent research. Biomarkers such as pregnancy-associated plasma protein A (PAPP-A), placental protein 13 (PP13), cell-free DNA (cfDNA), sex-hormone binding globulin (SHBG), follistatin, and myostatin show promise in predicting GDM, with varying sensitivities [64,65]. As counterparts, soluble fms-like tyrosine kinase (sFlt-1) and placental growth factor (PlGF) are integral to the angiogenic profile of preeclampsia and are effective indicators of placental dysfunction [66]. These biomarkers can predict the onset of clinical signs and symptoms, which will facilitate risk assessment and timely intervention for affected mothers and their fetuses. Despite this, current studies underscore the necessity of validating these findings through larger, prospective studies with standardized methodologies.

Currently, several crucial molecules involved in preeclampsia and FGR have emerged as prominent biomarkers in various studies. These include 1-deoxyspinglolipids (1-deoxySLs: 1-deoxysphinganine and 1-deoxysphingosine), polyamines (spermine and spermidine), and enzymatic markers (ornithine decarboxylase, spermidine/spermine N1-acetyltransferase, and spermine oxidase) [67]. The incorporation of these biomarkers into clinical settings holds promise for augmenting diagnostic precision regarding placental dysfunction. However, prospective validation studies are necessary to establish their clinical utility and further clarify their roles in the pathogenesis of conditions such as preeclampsia and FGR. Research into these biomarkers is advancing our understanding of the complex metabolic alterations associated with placental health and disease, paving the way for potential therapeutic strategies in the future.

### 5.5. Technological Advances

Innovative technologies are transforming the study of placental health. The introduction of AI-driven tools like HAPPY enables comprehensive analysis of placental histopathology, allowing for rapid assessment of cellular interactions and structural integrity. This technology enhances our ability to predict complications related to placental health, thereby improving maternal and infant care. Such advancements highlight the growing recognition of the placenta’s role in pregnancy and the need for detailed investigation into its biology [68].

## 6. Good Practices for Healthy Placental Function

Public policies related to maternal and fetal health significantly influence neonatal growth and development. These policies encompass a range of healthcare practices, monitoring protocols, and regulations regarding environmental exposures [30]. The following sections outline key areas where public policies affect fetoplacental and neonatal growth.

### 6.1. Clinical Management

Early detection of growth issues: Implementing routine ultrasound assessment to monitor placental development, fetal growth velocity, and the use of customized growth charts can enhance the detection of SGA infants. Early identification allows for timely interventions that improve outcomes [34]. Regular umbilical artery Doppler assessments are recommended for pregnancies at risk of FGR to monitor blood flow and make informed decisions regarding delivery timing [69].

Interventions for high-risk pregnancies: For pregnancies complicated by obesity or other risk factors, interventions such as low-dose aspirin have been shown to reduce the incidence of FGR by improving placental perfusion. Additionally, managing maternal health through lifestyle modifications, such as smoking cessation, is critical in preventing SGA outcomes [29].

### 6.2. Addressing Environmental Influences

Endocrine disruptors: Exposure to environmental EDCs such as BPA can negatively impact placental function and fetal growth [70]. Limiting exposure to these substances during pregnancy is essential for maintaining healthy fetoplacental dynamics.

Epigenetic considerations: Understanding the role of epigenetics in fetoplacental development can provide insights into how maternal stressors affect fetal outcomes [71]. Research suggests that epigenetic modifications may allow the fetus to adapt to adverse conditions at the expense of optimal placental function [72].

## 7. Gaps and Future Research Directions

Despite advancements in understanding placental dysfunctions, significant research gaps remain regarding the effects of various environmental factors and genetic influences on placental development. Public policies to fund research in these areas can help to bridge these gaps, leading to better-informed guidelines and practices supporting healthy fetal development [31].

Continued research into novel therapeutic strategies targeting placental function may offer new avenues for managing abnormal fetal growth patterns. They include exploring gene targeting and nanoparticle drug delivery systems to restore normal placentation and nutrient transfer. Investigating biomarkers for early detection of preeclampsia and other complications related to abnormal placentation could improve clinical outcomes. Creating predictive models based on machine learning can improve early detection and intervention efforts nationwide.

## 8. Conclusions

Placental-derived pregnancy complications remain a significant concern around the globe as well as in Mexico. The increasing prevalence of macrosomia highlights the need for targeted public health interventions to address underlying causes, such as maternal health and socioeconomic disparities. Along this line, the prevalence of FGR in Mexico is a multifaceted issue influenced by regional disparities and socioeconomic factors. Targeted interventions to improve maternal health education and access to prenatal care are essential for reducing the incidence of placental-derived pregnancy complications and its associated health risks in early infancy. Addressing these challenges requires a comprehensive approach that considers the unique needs of diverse populations across the country.

A holistic approach that addresses maternal health before conception and throughout pregnancy is needed to improve pregnancies and infant outcomes. This includes improving maternal nutrition and managing metabolic conditions, as well as ensuring optimal placental function. Community-based interventions promoting preconception care and maternal education have shown promise in improving health outcomes for mothers and their infants. By focusing on these interrelated factors, healthcare providers can better support women during their reproductive years and ultimately improve pregnancy and neonatal outcomes.

Current public policies play a crucial role in shaping the environment for placental function through monitoring practices, regulation of harmful substances, accessibility to care, and support for research initiatives. As the significance of these factors increases, it is imperative to sustain efforts to refine policies that facilitate optimal outcomes for maternal and neonatal health. Enhancing healthcare accessibility and quality regarding fetal growth and development requires improving monitoring techniques, understanding placental functions better, and developing effective interventions. Finally, addressing these areas is essential for reducing the risks associated with placental-derived pregnancy complications, leading to better maternal and neonatal health outcomes.

Placental health is further constrained by methodological limitations, as the predominant focus on HICs often overlooks the experiences of vulnerable populations in the Global South. This geographical bias raises concerns about the generalizability of findings and the potential for epistemic injustices, which ignore the unique health challenges faced by marginalized communities. Moreover, observational biases stemming from self-reported data and the misclassification of Indigenous populations may further obscure the true landscape of health disparities. In Mexico, despite the implementation of reforms aimed at enhancing maternal health, challenges persist, particularly with regards to the availability of high-quality healthcare for Indigenous populations and those residing in rural areas. The centralization of healthcare services and modifications in insurance models have sparked concerns regarding the continuity of care and equitable access to maternal health services. In summary, the persistent disparities in healthcare access and outcomes highlight the necessity for more inclusive and representative research methodologies to effectively address the complications of placental-derived pregnancy complications in diverse settings.

## Abbreviations

The following abbreviations are used in this manuscript:

FGR	Fetal growth restriction
IUGR	Intrauterine growth restriction
GDM	Gestational diabetes mellitus
MRI	Magnetic resonance imaging
hCG	Human chorionic gonadotropin
hPL	Human placental lactogen
HICs	High-income countries
LMICs	Low- and middle-income countries
LGA	Large for gestational age
NICU	Neonatal intensive care unit
FM	Fetal macrosomia
SGA	Small for gestational age
AGA	Appropriate for gestational age
EDCs	Endocrine-disrupting chemicals
BPA	Bisphenol A
HIFs	Hypoxia-inducible factors
